# Supplementary material for: Botrytis cinerea Loss and Restoration of Virulence during In Vitro Culture Follows Flux in Global DNA Methylation
Source: Int J Mol Sci. 2022 Mar 11;23(6):3034. doi: 10.3390/ijms23063034 (PMC8948621; doi:10.3390/ijms23063034)
Supplement: Supplementary file 1 [file ijms-23-03034-s001.zip › Table S2.pdf]

Table S2. Average coverage statistics for bisulfite samples.

Sample	Mean Coverage	Std dev	Cytosine coverage	Cytosine coverage (>4x)	mC (>1x)
T1	54.9939	20.3428	92.55%	87.65%	10.99%
T8	33.1991	13.868	91.50%	81.07%	9.14%
T8P	45.0209	17.0144	93.42%	86.67%	10.63%
