# Supplementary material for: Botrytis cinerea Loss and Restoration of Virulence during In Vitro Culture Follows Flux in Global DNA Methylation
Source: Int J Mol Sci. 2022 Mar 11;23(6):3034. doi: 10.3390/ijms23063034 (PMC8948621; doi:10.3390/ijms23063034)
Supplement: Supplementary file 1 [file ijms-23-03034-s001.zip › Table S3.pdf]

Table S3. Genome mapping statistics from 9 bisulfite converted Botrytis samples. All samples were mapped to *B. ciniera* B05.10 genome using Bismark/Bowtie2.

			Total methylated C's			Total non-methylated C's			% methylation			%mC
Samples	Total bp	% Map. Effic	CpG	CHG	CHH	CpG	CHG	CHH	CpG	CHG	CHH	
T1BS1	26382612	67.6	477254	438983	2499975	130600424	111869938	391807290	0.36	0.39	0.63	0.54
T1BS2	34048060	62.9	526341	485477	2494454	155155501	134341879	485255169	0.34	0.36	0.51	0.45
T1BS3	17347608	61.1	306505	279816	1575956	77226256	66054702	232502707	0.40	0.42	0.67	0.57
AvT1	25926093	63.87	436700	40	2190128	120994060	104088839	369855055	0.36	0.38	0.59	0.52
T8BS1	18543427	62	394348	360641	1568745	83351098	71894819	257263175	0.47	0.50	0.61	0.56
T8BS2	12613134	60.7	248968	229853	902624	55786373	47759672	166229028	0.44	0.48	0.54	0.51
T8BS3	17117307	65.1	391446	360065	2142019	79537475	69135859	257486354	0.49	0.52	0.83	0.71
AvT8	16091289	62.60	344920	316853	1537796	72891648	62930116	226992852	0.47	0.50	0.67	0.59
T8PBS 1	28336888	62.3	524875	487874	3561078	126943754	110309124	404488048	0.41	0.44	0.87	0.71
T8PBS 2	18471780	66.9	435528	403984	2932991	89352607	77332840	279892313	0.49	0.52	1.04	0.84
T8PBS 3	14694194	54.3	220458	201667	924438	57483914	49293008	173794940	0.38	0.41	0.53	0.48
AvT8P	20500954	61.17	393620	364508	2472835	91260091	78978324	286058433	0.43	0.46	0.86	0.68
