# Supplementary material for: Botrytis cinerea Loss and Restoration of Virulence during In Vitro Culture Follows Flux in Global DNA Methylation
Source: Int J Mol Sci. 2022 Mar 11;23(6):3034. doi: 10.3390/ijms23063034 (PMC8948621; doi:10.3390/ijms23063034)
Supplement: Supplementary file 1 [file ijms-23-03034-s001.zip › Table S8.pdf]

Table S8. Primer sequences used for MSAP.

**Oligo name**	**Function**	**Sequence**
Ad *Hpa*II/*Msp*I	Reverse Adaptor	GACGATGAGTCTAGAA
Ad. *Hpa*II/*Msp*I	Forward Adaptor	CGTTCT AGACTCATC
Ad. *EcoR*I	Reverse Adaptor	AATTGGTACGCAGTCTAC
Ad *EcoR*I	Forward Adaptor	CTCGTAGACTGCGTACC
Pre. *EcoR*I	Preselective primer	GACTGCGTACCAATTCA
Pre. *Hpa*II/*Msp*I	Preselective primer	GATGAGTCCTGAGCGGC
*EcoR*I2	Selective primer	GACTGCGTACCAATTCAAC
*Hpa*II 2.1	Selective primer	GATGAGTCCTGAGCGGCA
