# Supplementary figures and images for: Botrytis cinerea Loss and Restoration of Virulence during In Vitro Culture Follows Flux in Global DNA Methylation
Source: Int J Mol Sci. 2022 Mar 11;23(6):3034. doi: 10.3390/ijms23063034 (PMC8948621; doi:10.3390/ijms23063034)

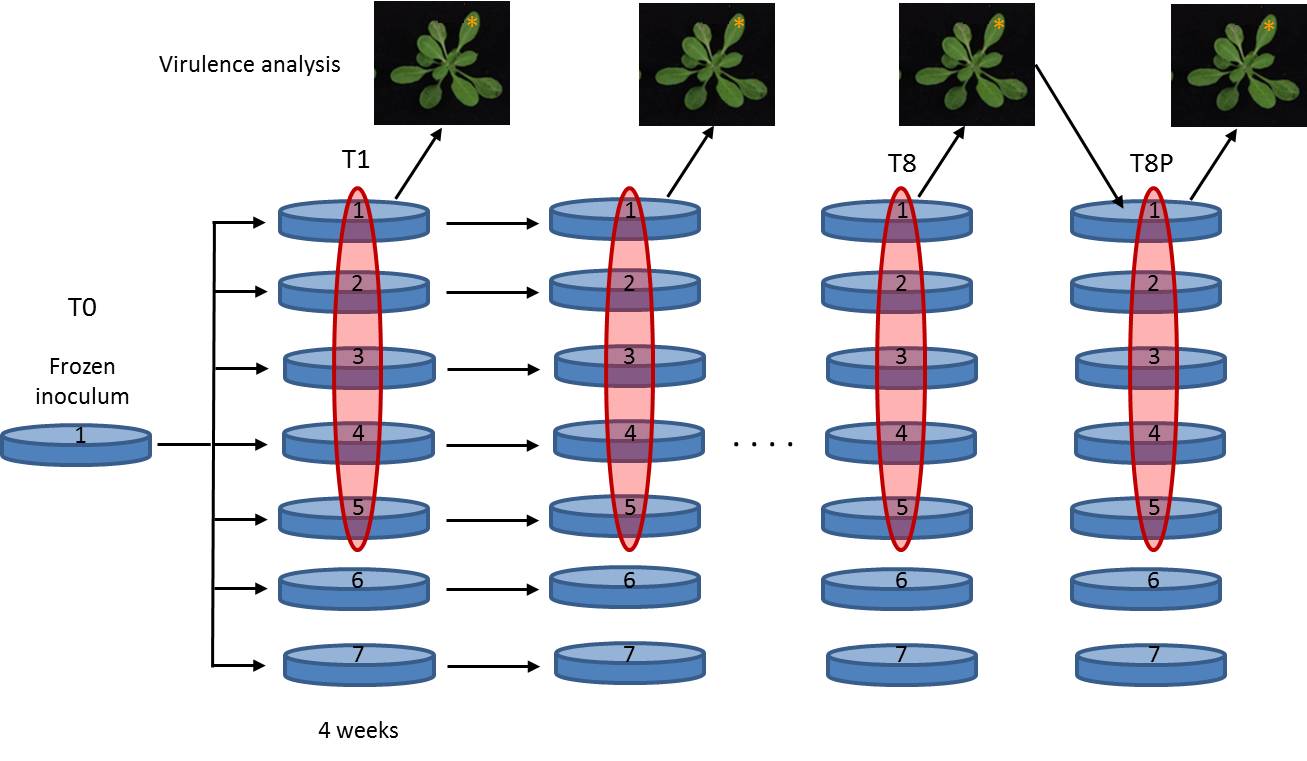

Supplement: Supplementary file 1 [file ijms-23-03034-s001.zip › Figure S1.jpg]
